# Silencing of long chain noncoding RNA paternally expressed gene (PEG10) inhibits the progression of neuroblastoma by regulating microRNA-449a (miR-449a)/ribosomal protein S2 (RPS2) axis

**DOI:** 10.1080/21655979.2022.2042999

**Published:** 2022-02-25

**Authors:** Jian Zhang, Wei Liu, Ping Ji, Yan Zhang

**Affiliations:** a Department of Geriatrics, Qingdao Chengyang District People’s Hospital, Qingdao, Shandong, P.R. China; b Department of Health Management, Qingdao Eighth People’s Hospital, Qingdao, Shandong, P.R. China; c Department of Ophthalmology, Qingdao Eighth People’s Hospital, Qingdao, Shandong, P.R. China; d The Third Department of Internal Medicine, Qingdao Women and Children’s Hospital, Qingdao University, Qingdao, Shandong, P.R. China

**Keywords:** LncRNA PEG10, neuroblastoma, miR-449a, RPS2

## Abstract

To investigate the mechanism of paternally expressed gene (PEG10) in regulating neuroblastoma (NB) progression. PEG10 expression was detected using quantitative real-time reverse transcription polymerase-chain reaction (qRT-PCR). The interaction of miR-449a and PEG10 or ribosomal protein S2 (RPS2) was employed by starBase, and then proved through RIP and dual-luciferase reporter assays. The NB cell viability, proliferation, invasion, and migration were evaluated by Cell Counting Kit-8 (CCK-8), colony formation, and Transwell assay. The mRNA and protein levels were determined by qRT-PCR and Western blotting, respectively. The levels of PEG10 and RPS2 were remarkably increased in NB tissues and cells, nevertheless the expression of miR-449a was conspicuously declined in NB tissues and cells. Silencing of PEG10 inhibited proliferation, migration, and invasion in SK-N-BE (2) cells, while overexpression of PEG10 promoted proliferation, migration, and invasion in SH-SY5Y cells. We affirmed that PEG10 interacted with miR-449a, and miR-449a could target the 3ʹUTR of RPS2 and negatively regulate its expression in NB cells. The upregulation of miR-449a inhibited proliferation, migration, and invasion in SK-N-BE (2) cells, while downregulation of miR-449a promoted proliferation, migration, and invasion in SH-SY5Y cells. Moreover, miR-449a overexpression weaken the function of PEG10-mediated on promoting proliferation, migration, and invasion in SH-SY5Y cells, while RPS2 overexpression rescued the effects of miR-449a-mediated on inhibiting those behaviors of SH-SY5Y cells. In conclusion, Silencing of PEG10 could inhibit proliferation, migration, and invasion via the miR-449a/RPS2 axis in NB cells.

## Graphical Abstract

**Figure:**
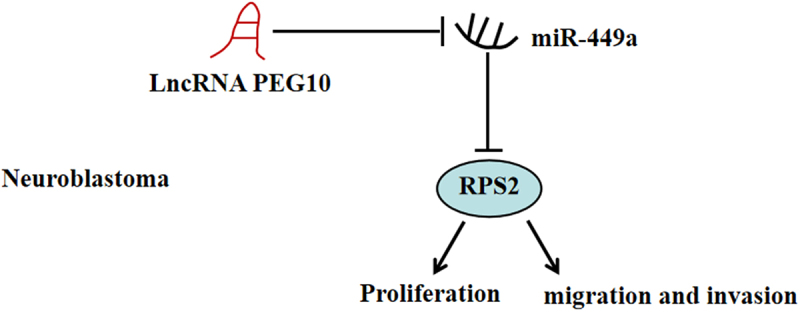

## Introduction

Neuroblastoma (NB) is a high-risk tumor type originating from the sympathetic nervous system or primitive neural crest. As extracranial tumor, it is most common in children, occupying about 15% of all child tumor-related mortality, threatening the health safety and life quality of children globally [1,2]. NB is featured by rapid cell proliferation, high degree of malignancy, strong heterogeneity, and most commonly invading lymph nodes, bone, and medulla ossium [3]. Although, some progresses have been made in clinical surgery, chemotherapy, radiotherapy, immunological therapy, stem cell transplantation and multidisciplinary intensive treatment, the overall survival rate of NB patients has not been significantly improved, especially in children with high-stakes clinical phenotype the long-term survival rate is less than 40% [4,5]. Therefore, studying the potential mechanism of NB progression is of great significance to find new early diagnosis and treatment targets.

Long-chain noncoding RNAs (lncRNAs) are a series of noncoding RNA nucleotides with a length of more than 200 bp, they are evolutionarily conservative and have epigenetic features that resemble to protein coding genes, but do not have the function of coding proteins [6]. Recently, it is estimated that about 15,000 lncRNAs have been found in the human genome [7]. Along with rapid large-scale genomics development, increasing evidence have manifested lncRNAs are abnormally expressed in different cancers and involve in a wide range of biological processes, such as cell metabolism, proliferation, differentiation, apoptosis, metastasis, and invasion [^8–10^]. Recently, some lncRNAs have been identified as new regulatory factors for the occurrence and development of NB, such as neuroblastoma highly expressed 1, DLX6 antisense RNA 1 [11,12], histocompatibility leukocyte antigen (HLA) Complex P5 [13], KCNQ1 opposite strand/antisense transcript 1 [14]. This study paid attention to the lncRNA paternally expressed gene 10 (PEG10) that firstly proposed in 2001 by Ono R et al [15]. The results of University of California Santa Cruz database reveal that lncRNA PEG10 (NONCODE Gene ID NONHSAG048235) is located between 94,285,681 and 94,298,949 base sites in human chromosome 7, with a total length of 763 bp. Many studies have shown that the abnormal expression of PEG10 is closely related to a variety of malignant tumors, such as bladder cancer [16], diffuse large B cell lymphoma [17], hypopharyngeal squamous cell carcinoma [18], esophageal cancer [19]. However, the specific role of PEG10 in human NB is still unclear.

MicroRNAs (miRNAs) are non-coding RNAs (~22nts), which participate in gene regulation at the post-transcriptional level and play a crucial part in many biological behaviors of cancer. Recently, the imbalance of miR-449a was found in NB, which plays an anti-cancer role by inducing cell differentiation and cell cycle arrest [20]. Some lncRNAs usually act on cancer cells via regulating miRNA, including PEG10 [21]. For example, Zhao et al. have reported that PEG10 regulated cell proliferation, apoptosis, and metastasis through miR-101-3p/Kinesin family protein 2A axis in diffuse large B-cell lymphoma [22]. So far, the role of PEG10 and miR-449a and their regulatory relationship in NB remain unclear.

Therefore, this study aimed to explore the role of PEG10 in the progression of NB. We hypothesize that PEG10 participates in the NB progression, which may be involved in the miR-449a/RPS2 axis.

## Materials and methods

### Patient specimens

Patients undergoing NB, who were treated at our hospital, were enrolled from January 2019 to November 2020. A total of 20 NB tumor tissues were obtained during surgical resection. The inclusion criteria for patient selection were as follows: 1. patients didn’t undergo radiotherapy or chemotherapy before surgery; 2. patients had no other malignant tumors. The exclusion criteria for patient selection were as follows: 1. patients didn’t sign the written informed consents; 2. patients had incomplete clinicopathological data. Human embryonic tissues or normal dorsal root ganglia tissues served as normal tissues, which were obtained from therapeutic abortions (at day 50 of gestation) and interrupted pregnancies. All tissue specimens were collected, quickly snap-frozen in liquid nitrogen and reposited at −80°C for further using. The Ethics and Research Committees of our hospital has ratified the study (approval number: QDFY20181106), and the written informed consents were completed by parents or legal guardians.

### Cell culture and transfection

Human umbilical vein endothelial cells (HUVEC) and NB cell lines [SH-SY5Y, SK-N-SH, SK-N-AS, and SK-N-BE (2)] were acquired from Procell (China). The cells were hatched in RPMI-1640 medium (Thermo Fisher Scientific, MA, USA) with 10% of fetal bovine serum (FBS; Hyclone, Logan, UT, US), placed in a constant-temperature incubator (Thermo Fisher Scientific,) under a relative humidity of 90% and a controlled atmosphere with 5% CO_2_ at 37°C. The small interfering RNA (siRNA) targeting LncRNA PEG10 (si-PEG10-1 and si-PEG10-2) and ribosomal protein S2 (RPS2; si-RPS2-1 and si-RPS2-2), LncRNA PEG10 or RPS2 overexpression plasmid (PEG10, RPS2), miR-449a mimic, miR-449a inhibitor, and their corresponding controls (si-NC, vector, mimic NC and inhibitor NC, respectively) were provided by Ribobio (Guangzhou, China) and transfected using Lipofectamine 3000 (Invitrogen, Carlsbad, CA), according to the manufacturer’s description. After 48 h transfection, the cells were collected for subsequent experiments.

### Quantitative real-time reverse transcription polymerase chain reaction (qRT-PCR)

The TRIzol reagent (Invitrogen) was used to extract total RNA from cells and tissues on the basis of the description of manufacturer. Then, 500 ng total RNA was applied for reverse transcription by PrimeScript RT reagent Kit Perfect Real-Time kit (Takara, Japan). After cDNA was amplified by a real-time PCR kit (Takara), qRT-PCR was performed to detect PEG10, miR-449a, or RPS2 expressions by SYBR PremixEx Taq II kit and PCR Master Mix (Applied Biosystems, USA) on a 7500 Real-Time PCR System (Applied Biosystems, USA). The concentration and purity of total RNA and cDNA were evaluated using NanoDrop 1000 Spectrophotometer (USA). The PCR reaction system was as followed: 10 μl PCR Master Mix, 0.4 μl reverse primer, 0.4 μl forward primer, 1 μl cDNA, 0.4 μl SYBR PremixEx Taq II, 7.8 μl ddH_2_O. The reactive conditions were as following: predenaturation, 95°C, 3 min; denaturation, 96°C 15 sec; and annealing, 58°C, 30 sec, 40 cycles. The sequences of primers were as followed: lncRNA PEG10: forward 5’-TCATTCCCCGTGCTTATCGG-3’, reverse 5’-TTCCACTCCAATCAGTGCCC-3’; miR-449: forward 5’-GCGGCGGTGGCAGTGTATTGTTAG-3’, reverse 5’- ATCCAGTGCAGGGTCCGAGG-3’; RPS2: forward 5’-CCGAGGATAAGGAGTGGATGC-3’, reverse 5’-CCCCGATAGCAACAAATGCC-3’; glyceraldehyde-3-phosphate dehydrogenase (GAPDH): forward 5’-GGATTTGGTCGTATTGGGCG-3’, reverse 5’-TCCCGTTCTCAGCCATGTAGT-3’; U6: forward 5’-CAGCACATATACTAAAATTGGAACG-3’, reverse 5’-ACGAATTTGCGTGTCATCC-3’. The relative expressions of lncRNA PEG10, RPS2, and miR-449 were calculated using the 2^−ΔΔCt^ method and normalized to GAPDH and U6 [23].

### Subcellular fractionation

According to the manufacturer’s instructions, PARIS Kit (Life Technologies, USA) was used to perform nuclear and cytoplasmic separation. In brief, SK-N-BE (2) and SH-SY5Y cells (5 × 10^6^ cells) were resuspended in 600 μl resuspension buffer for 15 min. The cytoplasmic fraction was obtained from the supernatant content after centrifugation at 400 × g for 15 min. Next, the pellet was resuspended in 300 μl PBS, nuclear isolation buffer, and RNase-free H_2_O for 20 min. After centrifugation, the pellet underwent nuclear fractionation. The expression of PEG10, GAPDH, and U6 was determined by qPCR [24].

*Cell Counting Kit-8* (*CCK-8) assay*

The viability of cells cultured for 1, 2, 3, and 4 days was evaluated by CCK-8 (GenePharma, Shanghai, China). In detail, the transfected SK-N-BE (2) and SH-SY5Y (both 5 × 10^4^ cells/well) were seeded in 96-well plates with 100 μl medium in each well and cultured at 37°C. Next, 10 μl CCK-8 solution was added into per well and hatched at 37°C for 4 h. Finally, the absorbance at 450 nm was measured by a microplate reader (BioTek Instruments, US) [25].

### Colony formation assay

After transfection for 48 h, the transfected SK-N-BE (2) and SH-SY5Y cells at a density of 400 cells/well were seeded in 6-well plate with complete medium and incubated for 2 weeks at 37°C incubator with 5% CO_2_. The colonies with greater than 50 cells were then fixed with 4% formaldehyde for 15 min, stained with crystal violet for 10–20 min, lastly counted under the microscope. Finally, the data from five stochastic fields were performed to statistics [26].

### Transwell assay

The migration and invasion capabilities of transfected NB cells was assessed using Transwell chambers with 8 μm porous membranes coated without or with Matrigel (BD Biosciences). The transfected SK-N-BE (2) and SH-SY5Y cells (both 5 × 10^5^ cells/mL) in RPMI-1640 medium were seeded into the upper chamber. Followed, 500 μL RPMI-1640 medium adding 10% FBS was appended to the lower chamber. 48 h later, the cells on the upper side of the membrane were removed using a cotton swab, followed by multiple times PBS washing. In quick succession, the cells on the lower surface of the membrane were fixed with 4% paraformaldehyde solution for 20 min, and stained with 0.2% crystal violet (Biotechnology, Shanghai, China) for 15 min. Ultimately, an inverted microscope (Nikon, Tokyo, Japan) was used to capture images, and the cells were counted [27].

### Western blotting

SK-N-BE (2) and SH-SY5Y cells were lysed in RIPA buffer (Beyotime Biotechnology, China) with protease and phosphatase inhibitors for 30 min on ice. The protein concentration was tested via a BCA Protein Assay kit (Beyotime Biotechnology). The proteins were separated by 10% sodium dodecyl sulfate-polyacrylamide gel electrophoresis and transfered to polyvinylidene fluoride membrane (Millipore, USA). Then, the membrane were clogged with 5% nonfat dry milk, and incubated with primary antibodies [matrix metalloproteinase-2 (MMP-2), MMP-9, MMP-14, RPS2, and GAPDH; 1:1000, Abcam, Cambridge, MA] at 4°C for overnight. After phosphate buffer saline washing, the membranes were incubated with horseradish peroxidase-labeled secondary antibody (1:2000, Cell Signaling, USA) for 2 h indoor temperature. Ultimately, intensity of protein expression was measured by an enhanced chemiluminescence reagent (Thermofisher, USA). GAPDH served as control.

### Luciferase reporter assay

The bioinformatics software StarBase (https://starbase.sysu.edu.cn/) was used to predict the potential-binding sites of PEG10-miRNA interactions and miR-449a-miRNA interactions. The target interactions between miR-449a and PEG10 or RPS2 were confirmed by Dual-luciferase reporter assay. The predictive wild type (WT) of binding sequences with miR-449a in PEG10 and RPS2 were amplified and cloned into pmirGLO vector (Promega), called as PEG10-WT and RPS2-WT. Simultaneously, the mutant type (MUT) of binding sequences in PEG10 and RPS2 were also synthesized by site-directed gene mutagenesis kit (Takara, Japan) and inserted into pmirGLO vector (Promega), named as PEG10-MUT and RPS2-WT. SK-N-BE (2) and SH-SY5Y cells were inoculated into 24-well plates for 24 h, then transfected with 50 ng of reporter plasmids along with 20 nM of miR-449a or mimic NC with Lipofectamine 3000 (Invitrogen). After transfection for 48 h, cells were lysed, luciferase activities were assessed by a Dual-Luciferase Reporter Assay Kit (Promega) in light of the instructions of manufacturer and normalized with Renilla luciferase as control [28].

### Statistical Analysis

All data were analyzed by SPSS 20.0 package (Chicago, IL, USA). All above experiments were conducted in triplicate and all data were expressed as mean ± standard deviation (SD). The normal distribution of data was analyzed using Kolmogorov–Smirnov test. Differences between two groups were determined by Student’s t-test, differences among multiple groups were determined by analysis of variance (ANOVA). The relationship between the PEG10 level and clinicopathological parameters of NB patients were analyzed using the chi-square test. P < 0.05 was regarded as statistically significant.

## Results

In this study, the aim was to explore whether PEG10 could influence NB including cell proliferation, migration, and invasion and investigate the molecular mechanism of PEG10 in NB progression. We found that PEG10 was highly expressed in NB tissues and cells. Silencing of PEG10 inhibited NB cell proliferation, migration, and invasion via regulating the miR-449a/RPS2 axis.

### LncRNA PEG10 was upregulated in human NB tissues and cell lines

**Figure 1.**
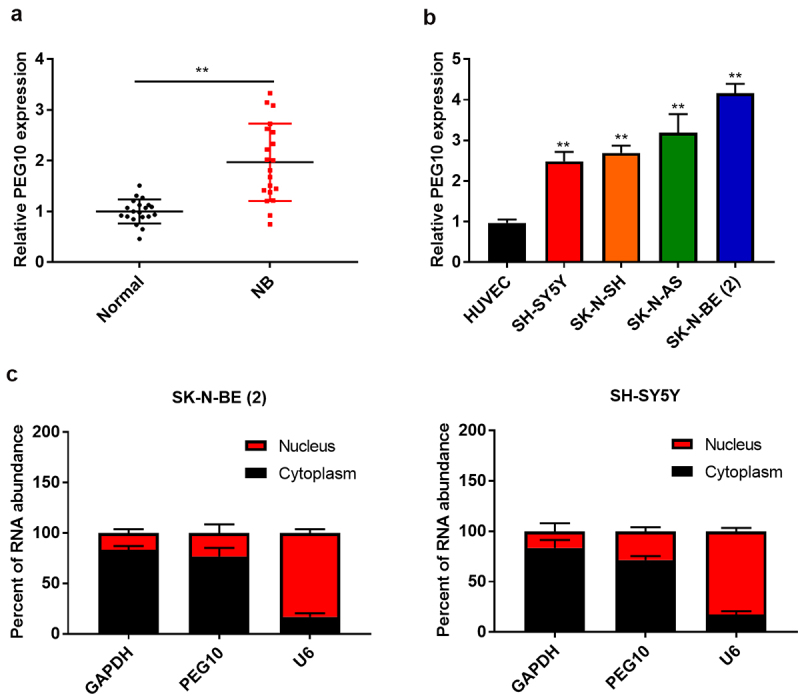
LncRNA PEG10 expression was up-regulated in NB tissues and cells. (a) PEG10 expression in NB and normal tissues was detected by RT-QPCR. (b) PEG10 expression was measured in HUVEC and NB cells [SK-N-SH, SH-SY5Y, SK-N-AS, SK-N-BE (2)] by RT-qPCR. (c) Subcellular fractionation for PEG10 in SK-N-BE (2) and SH-SY5Y cells. Compared with normal tissues or HUVEC cells:*P < 0.05; **P < 0.01.

To explore the potential involvement of PEG10 in NB progression, we analyzed the expression level of PEG10 in 20 pairs of NB tissues and adjacent normal tissues by qRT-qPCR. The results indicated PEG10 expression was obviously higher in NB tissues, compared to that in normal tissues (P < 0.05, Figure 1(a)). The NB patients were divided into two groups (n = 10 in each group) according to median of PEG10 expression. The results showed that the PEG10 levels was related to the INSS stage (P = 0.0246) and lymph node metastasis (P = 0.0062, Table 1). Afterward, PEG10 expression was also detected in NB cell lines and HUVEC cell line. As shown in Figure 1(b), compared to HUVEC cells, PEG10 were remarkably upregulated in NB cell lines (SH-SY5Y, SK-N-SH, SK-N-AS, and SK-N-BE (2) (P < 0.05). These results indicate that PEG10 may be related to NB progression. Then, nuclear-cytoplasmic fractionation assay showed that PEG10 was mainly located within the cytoplasm in SH-SY5Y and SK-N-BE (2) cells (Figure 1(c)).

**Table 1.** Relationship between PEG10 expression and clinicopathologic characteristics of neuroblastoma patients

Group	Case	PEG10 expression		P value
Gender		Low	High	0.3613
Male	8	5	3	
Female	12	5	7	
Age(year)				0.3291
<3	14	6	8	
≥3	6	4	2	
Tumor diameter (cm)				0.1213
<3	15	6	9	
≥3	5	4	1	
INSS stage	15			0.0246*
1, 2, 4S	11	8	3	
3, 4	9	2	7	
Lymph node metastasis				0.0062**
Yes	12	9	3	
No	8	1	7

### Silencing of LncRNA PEG10 suppressed the proliferation, migration, and invasion of NB cells

**Figure 2.**
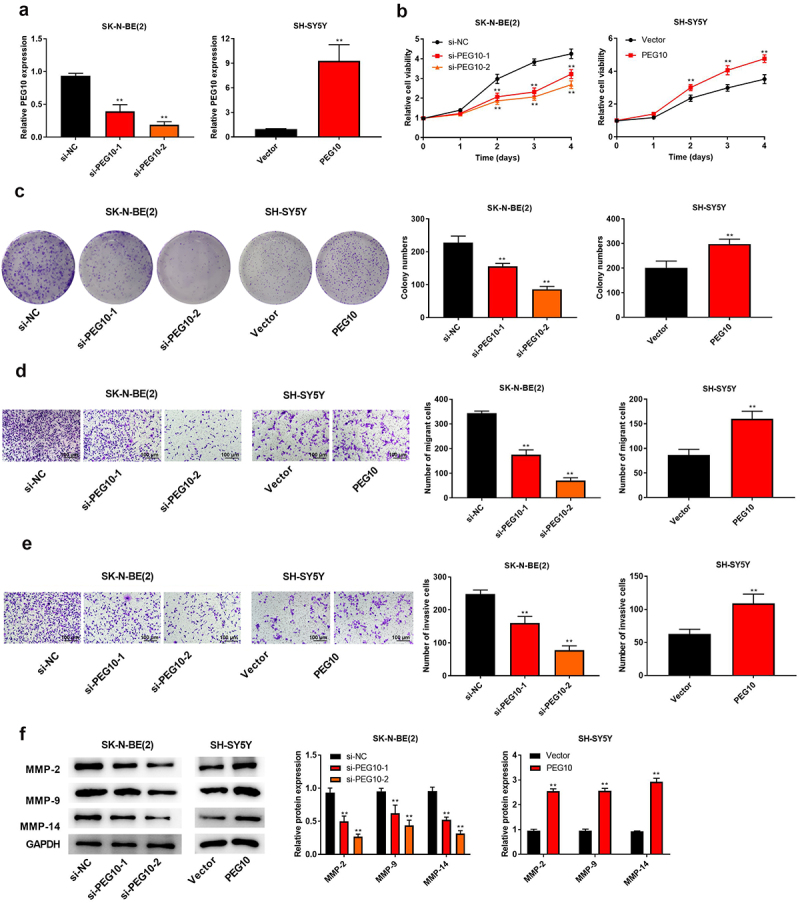
LncRNA PEG10 silencing inhibited NB cell proliferation, migration and invasion. (a) PEG10 expression in SK-N-BE (2) and SH-SY5Y cells was tested by qRT-PCR. (b) The activity of SK-N-BE (2) and SH-SY5Y cells was measured by CCK-8 assay. (c) The proliferation of SK-N-BE (2) and SH-SY5Y cells was detected via colony formation assay. (d) Transwell assay was used to detect the migration of SK-N-BE (2) and SH-SY5Y cells. (e) Transwell assay was used to detect the invasion of SK-N-BE (2) and SH-SY5Y cells. (f) The protein levels of MMP-2, MMP-9 and MMP-14 in SK-N-BE (2) and SH-SY5Y cells were detected by Western blotting. **P < 0.01, compared with si-NC or vector group.

To verify the specific functions of PEG10 on biological behaviors of NB cells, SK-N-BE(2) and SH-SY5Y cells, which respectively expressed highest and lowest PEG10, were selected to conduct functional experiments. The si-PEG10-1 and si-PEG10-2 were designed to knockdown PEG10 in SK-N-BE (2) cells, and PEG10 plasmid was to construct PEG10 overexpression in SH-SY5Y cells, and the expression level of PEG10 was verified using qRT-PCR (Figure 2(a)). Afterward, CKK-8 and colony formation assays were used to detect the influence of PEG10 on cell viability and proliferation, respectively. It was discovered that PEG10 knockdown suppressed cell proliferation and colony number of SK-N-BE (2) cells (P < 0.05), whereas PEG10 overexpression indicated promoting effects on SH-SY5Y cells (Figure 2(b-c)). Further, Transwell assay was implemented to confirm the impacts of PEG10 on cell migration and invasion. As shown in Figure 2(d-e), PEG10 knockdown prominently decreased the number of SK-N-BE (2) cell migration and invasion (P < 0.05), whereas the contrary effect was observed when PEG10 was overexpressed in SH-SY5Y cells. In addition, silencing of PEG10 straightforwardly increased the expressions of MMP2, MMP9, and MMP14 in SK-N-BE (2) cells (P < 0.05, Figure 2(f)). Conversely, PEG10 overexpression had the opposite effects on these proteins in SH-SY5Y cells (Figure 2(f)). All those results implied that silencing of LncRNA PEG10 pronouncedly impeded NB cells to proliferate, migration, and invasion.

### LncRNA PEG10 negatively regulated the expression of miR-449a

**Figure 3.**
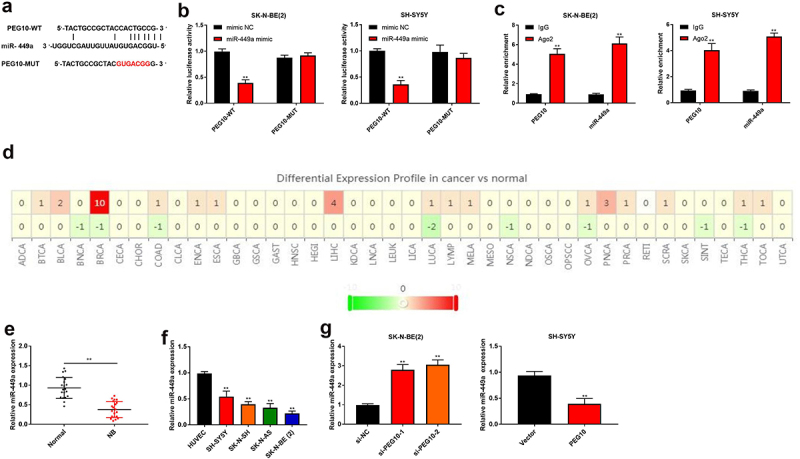
LncRNA PEG10 negatively regulated the expression of miR-449a. (a) Binding sites of PEG10 to miR-449a. (b) Luciferase activity was detected by Dual-luciferase reporter to verify the binding of PEG10 to miR-449a. (c) RIP assay was used to verify the binding of PEG10 to miR-449a. (d) MiR-449a expression in generalized carcinoma was analyzed by the Cancer Genome Atlas (TCGA) database. (e) QRT-PCR was used to test miR-449a expression in NB and normal tissues. (f) MiR-449a expression was measured in HUVEC and NB cells [SK-N-SH, SH-SY5Y, SK-N-AS, SK-N-BE (2)] by RT-qPCR. (g)The miR-449a expression in SK-N-BE (2) and SH-SY5Y cells was detected by qRT-PCR. **P < 0.01, compared with IgG, mimic NC, Normal, si-NC, or vector group.

To study the mechanism of PEG10 in NB, the downstream target of PEG10 were searched. As shown in Figure 3(a), miR-449a was predicted as a latent downstream target of PEG10 through bioinformatic analysis software StarBase. Luciferase reporter assay indicated that miR-449a mimic’s transfection weakened the luciferase activity in SK-N-BE (2) (P < 0.05) and SH-SY5Y cells that were transfected with PEG10-WT, nevertheless no significant variation was observed in the PEG10-MUT group (Figure 3(b)). Moreover, RIP assay showed that the enrichment of PEG10 and miR-449a in Ago2 precipitate was higher than that to IgG (P < 0.05, Figure 3(c)). The Cancer Genome Atlas (TCGA) database showed that miR-449a in many tumor tissues were downregulated (Figure 3(d)). Afterward, the expression of miR-449a in NB tissues, cells and the transfected NB cells was assessed by qRT-PCR. The result showed that the level of miR-449a was conspicuously decreased in NB tissues and cells (Figure 3(e,f)). Further finding indicated that miR-449a expression could be upregulated by si-PEG10-1 and si-PEG10-2 in SK-N-BE (2) cells (P < 0.05), while miR-449a expression could be downregulated by PEG10 overexpression in SH-SY5Y cells (P < 0.05, Figure 3(g)). Collectively, the above results indicated PEG10 could directly bind to miR-449a and negatively regulate its expression in NB cells.

### The upregulation of miR-449a inhibited the proliferation, migration, and invasion of NB cells

**Figure 4.**
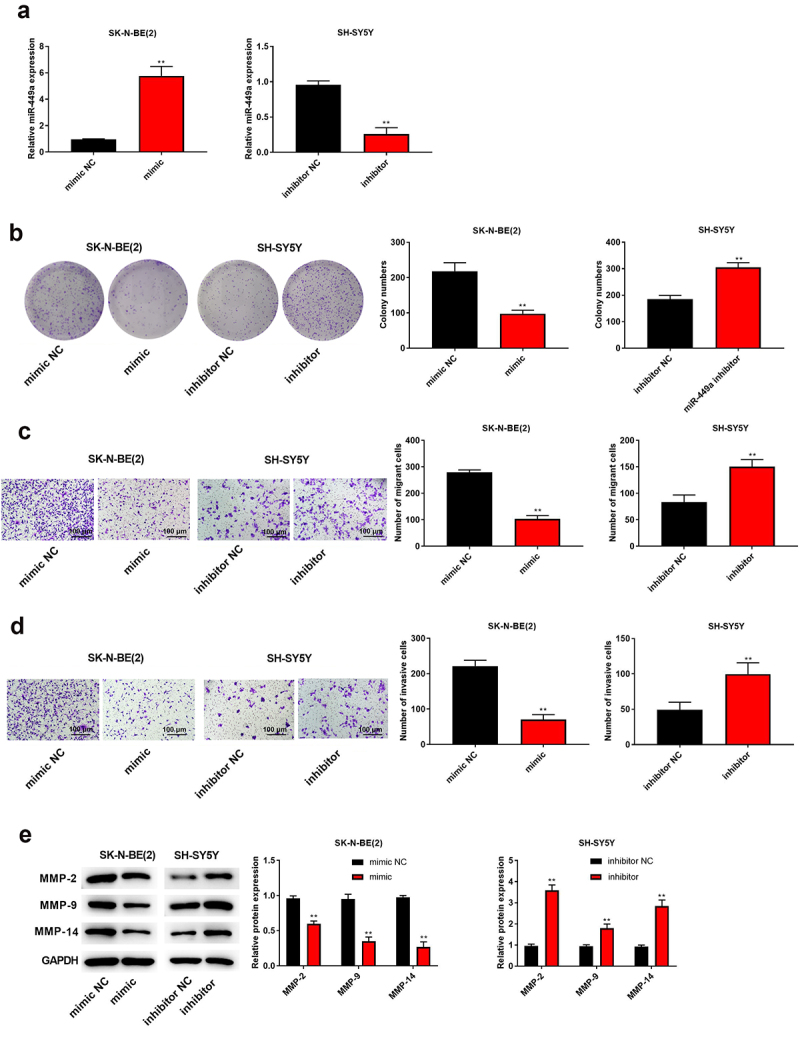
High expression of miR-449a inhibited the proliferation, migration and invasion of NB cells. (a) The miR-449a expression in SK-N-BE (2) and SH-SY5Y cells was tested via qRT-PCR. (b) The proliferation of SK-N-BE (2) and SH-SY5Y cells was tested via colony formation assay. (c) The migration ability of SK-N-BE (2) and SH-SY5Y cells was tested through Transwell assay. (d) The invasive ability of SK-N-BE (2) and SH-SY5Y cells was detected by Transwell assay. (e) The protein levels of MMP-2, MMP-9, and MMP-14 in SK-N-BE (2) and SH-SY5Y cells were detected by Western blotting. **P < 0.01, compared with mimic NC or inhibitor NC group.

To detect the biological role of miR-449a in NB, SK-N-BE(2) and SK-SY5Y cells were transfected with miR-449a mimic/mimic NC and miR-449a inhibitor/inhibitor NC, respectively. RT-qPCR results demonstrated that miR-449a expression was observably upregulated in SK-N-BE (2) cells (P < 0.05), while miR-449a expression was downregulated in SK-SY5Y cells after transfection (P < 0.05, Figure 4(a)). Moreover, upregulation of miR-449a suppressed the colony-forming, invasive and migrative abilities of SK-N-BE (2) cells (P < 0.05), while downregulation of miR-449a has opposite effects on those abilities of SK-SY5Y cells (Figure 4(b-d)). Meanwhile, upregulation of miR-449a straightforwardly decreased the expression of MMP2, MMP9, and MMP14 in SK-N-BE (2) cells (P < 0.05), whereas downregulation of miR-449a manifested promoting effects on expression of these proteins in SH-SY5Y cells (Figure 4(f)). These findings manifested that upregulation of miR-449a could restrain the proliferation, migration, and invasion of NB cells.

### MiR-449a negatively regulates RPS2 expression

**Figure 5.**
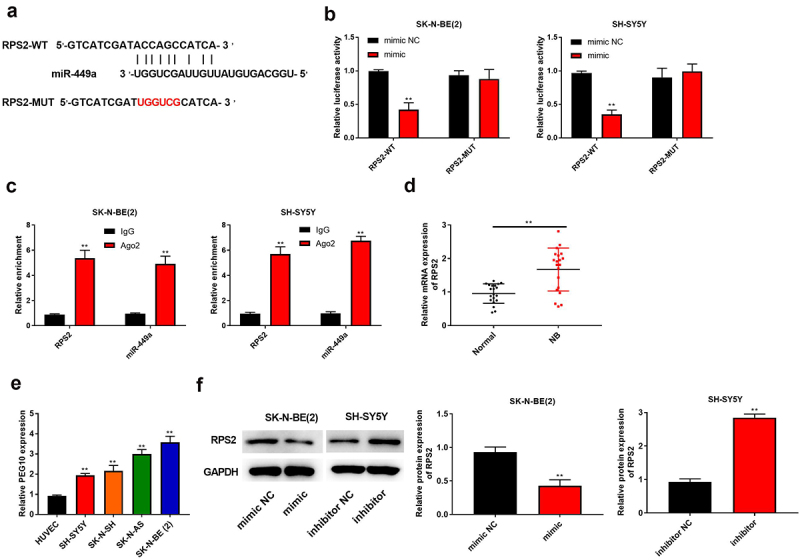
MiR-449a negatively regulated RPS2 expression. (a) The binding site of RPS2 and miR-449a. (b) Luciferase activity assay was used to verify the binding of RPS2 to miR-449a. (c) RIP assay was used to verify the binding of RPS2 to miR-449a. (d) QRT-PCR was used to detect the expression of RPS2 in NB and normal tissues. (e) RPS2 expression was measured in HUVEC and NB cells [SK-N-SH, SH-SY5Y, SK-N-AS, SK-N-BE (2)] by RT-qPCR. (f) Western blotting was used to test RPS2 protein in SK-N-BE (2) and SH-SY5Y cells. **P < 0.01, compared with mimic NC, inhibitor NC or Normal group.

To inspect the hidden mechanism of miR-449a in NB cells, the online database of StarBase for bioinformatic analysis was performed, predicting miR-449a directly binds to RPS2 3’-UTR (Figure 5(a)). To confirm this prediction, luciferase reporter gene assay and RIP assay were used to validate. Data implied that the luciferase activity of RPS2-WT was apparently decreased by miR-449a in SK-N-BE (2) (P < 0.05) and SH-SY5Y cells, nevertheless the activity of RPS2-MUT had no changing (Figure 5(b)). RIP assay showed that the enrichment of RPS2 and miR-449a in Ago2 precipitate was higher than that to IgG (P < 0.05, Figure 5(c)). Subsequently, the expression of RPS2 in human NB tissues, cells and transfected SK-N-BE (2) and SH-SY5Y cell lines were detected by RT-qPCR and Western blot analysis, respectively. The results indicated that RPS2 was apparently upregulated in human NB tissues and cells (P < 0.05, Figure 5(d-e)). RPS2 expression could be downregulated by miR-449a mimic in SK-N-BE (2) cells (P < 0.05), while it be upregulated by miR-449a inhibitor in SH-SY5Y cells (P < 0.05, Figure 5(f)). Collectively, miR-449a could directly bind to RPS2 and negatively regulate its expression in NB cells.

*Silencing of LncRNA PEG10 inhibited NB cell proliferation, migration, and invasion by regulating miR-449a/RPS2*

**Figure 6.**
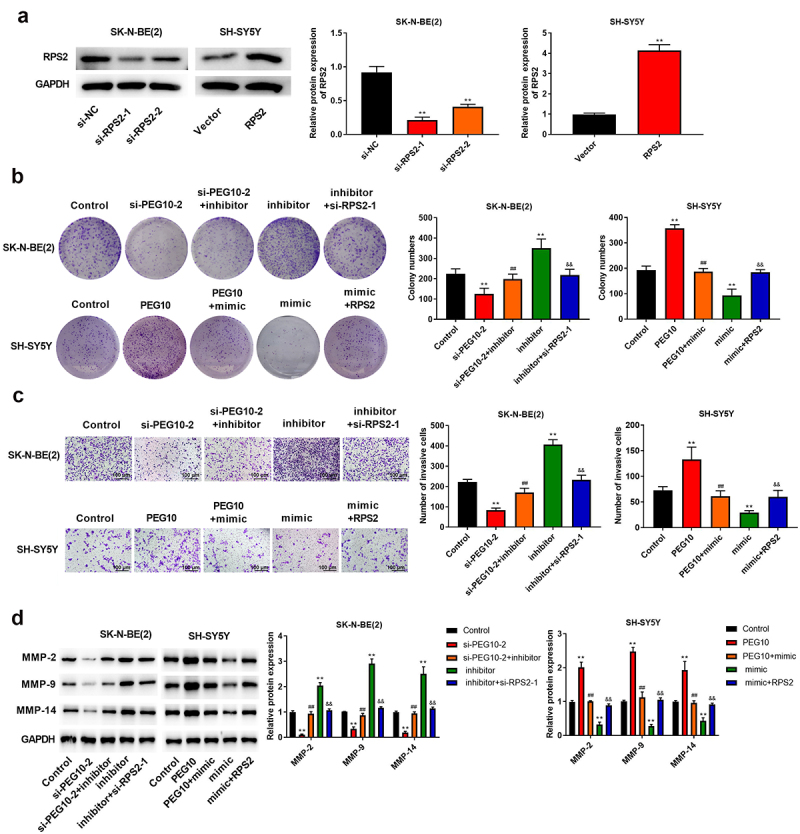
LncRNA PEG10 silencing inhibited the proliferation, migration, and invasion of NB cells via modulating the miR-449a/RPS2 axis. (a) RPS2 expression in SK-N-BE (2) and SH-SY5Y cells was tested using Western blotting. (b) The proliferation of SK-N-BE (2) and SH-SY5Y cells was tested via clone formation assay. (c) The invasion of SK-N-BE (2) and SH-SY5Y cells was detected by Transwell assay. (d) The protein expression of MMP-2, MMP-9, and MMP-14 in SK-N-BE (2) and SH-SY5Y cells were detected via Western blotting. **P < 0.01, compared with control group; ##P < 0.01, compared with si-PEG10-2 or PEG10 group; &&P < 0.01, compared with mimic or inhibitor group.

To explore whether the PEG10 promoted NB development by regulating miR-449a/RPS2 axis, we carried out some rescue experiments. Firstly, SK-N-BE (2) cells were transfected with si-RPS2-1, si-RPS2-2, or si-NC, SH-SY5Y cells were transfected with RPS2 or vector, the expression levels of RPS2 was verified using Western blotting (Figure 6(a)). Afterward, SK-N-BE(2) cells were transfected with si-PEG10-2, si-PEG10-2+ miR-449a inhibitor, miR-449a inhibitor, miR-449 inhibitor+si-RPS2-1; SH-SY5Y cells were transfected with PEG10, PFG10+ miR-449a mimic, miR-449a mimic, miR-449a mimic + RPS2. As expected, the number of colonies and invasive cells, as well as the expression levels of MMP-2/9/14 prominently inhibited in SK-N-BE(2) cells transfected with si-PEG10 (P < 0.05), which were partly recovered in cells transfected with si-PEG10+ miR-449a inhibitor, while si-RPS2 was noticeably abolished the regulatory effect of miR-449a inhibitor (Figure 6(b-d)). Likewise, the number of colonies and invasive cells, as well as the expression levels of MMP-2/9/14 were prominently increased in SH-SY5Y cells transfected with PEG10 (P < 0.05), which were partly reduced in cells transfected with PEG10+ miR-449a mimic, while RPS2 was notably weakened the regulatory effect of miR-449a mimic (Figure 6(b-d)). In brief, those data revealed that PEG10 silencing inhibited NB cells proliferation, migration, and invasion by regulating miR-449a/RPS2.

## Discussion

NB is with high incidence rate, especially in developed countries, about 10,0000–250,000 per million [29]. Recently, accelerating evidence have shown that lncRNA is closely associated with tumors, and their expression changes affect the initiation and progression of cancers [8]. LncRNA may regulate miRNA expression at various levels, such as chromatin modification, splicing, transcription, post-transcriptional regulation, translation, and small RNA processing, thereby affecting and regulating cell proliferation, differentiation, cycle, and apoptosis [30]. Therefore, exploring the formation mechanism of NB from the perspective of lncRNA may be helpful to deeply understand the pathogenesis of NB.

Some researchers have reported compared with corresponding normal samples, lncRNA PEG10 was overexpressed in esophageal cancer [19], hypopharyngeal squamous cell carcinoma [18], diffuse large B-cell lymphoma [22], suggesting PEG10 may be a carcinogenic lncRNA. One study has shown that si-PEG10 could inhibit the viability, migration and invasion of human glioma cell line U251 and promote apoptosis by upregulating miR-506 [31]. In our study, the possible mechanism of PEG10 in NB cells was studied. Firstly, our study found that the expression level of PEG10 in NB tissue was significantly higher, which confirmed that PEG10 may be a valuable biomarker for the diagnosis of NB. Further, we also studied the biological function of PEG10 in NB cells. We found that downregulation of PEG10 inhibited NB cell proliferation, migration, and invasion. The repeated amplification of PEG10 and its ability to significantly increase tumor malignant behavior suggest that PEG10 may play a driving role in NB.

Emerging findings have indicated that lncRNA can action as ceRNA via sponging and secluding miRNAs in a specific sequence and weaken their impaction of binding and restraining target mRNAs. Utilizing the StarBase online tool, we firstly verified PEG10 obliged as a sponge of miR-449a, a low expressed miRNA in NB. MiR-449a has been proved to be dysregulated in multiple types of human cancers, such as lung cancer [32], prostate cancer [33], nasopharyngeal carcinoma [34]. Further, Zhao et al. have investigated the tumor-suppressive effect of miR-449a in NB, it was suggested that the differentiation-inducing effect of miR-449a played a key role in identifying the progression of NB [20]. In addition, some studies have demonstrated that couple of lncRNAs, such as lincRNA-p21 [35], LINC01106 [36], small nucleolar RNA host gene 7 [37], perform an increasingly vital role in carcinogeneses of human cancers through targeting miR-449a. In the present study, our findings advocated the antitumor peculiarity of miR-449a in NB, which is consistent with previous report of Zhao et al [20]. The more important concerning, we found for the first time that miR-449a was a functional regulator of PEG10 in modulating the malignant development of NB cells *in vitro*.

It is well known that microRNAs (miRNAs) take part in cancer development via regulating mRNA. RPS2 is a 32 kDa ribosomal protein, it is encoded by LLRep3 that belong to the member of highly conservative mammalian repeat gene family [38]. RPS2 is involved in ribosome-bound aminoacyl transfer RNA, which may affect the precision of mRNA translation. It has been reported that RPS2 promotes tumors, and it is overexpressed in human prostate cancer [38], breast cancer, squamous cell carcinoma [39], and hepatocellular carcinoma [40]. In addition, miRNAs target RPS2, such as pre-let-7a binding to RPS2 could block expression of let-7a, thereby promoting prostate tumor growth [41]. In our study, we confirmed that RPS2 was highly expressed in NB tissues, luciferase reporter gene assay and RIP assay showed that RPS2 was one of supervise downstream targets of miR-449a, and miR-449a inhibited RPS2 expression. In addition, miR-449a overexpression weakened the carcinogenic effect of PEG10, while the addition of RPS2 eliminated the inhibitory effect of miR-449a overexpression on the proliferation, migration, and invasion of NB cells, indicating that PEG10 regulating RPS2 expression by targeting miR-449a in NB cells.

However, there are some weaknesses in this study. Firstly, knockdown of PEG10 and overexpression of PEG10 were both carried out in SK-N-BE (2) and SH-SY5Y cells would be strengthened our study. Secondly, the animal experiment is needed to confirm the effects of PEG10. In the future study, we would further explore the effects of PEG10 on tumor growth through *in vivo* experiment.

## Conclusion

In summary, PEG10 expression was higher in NB tissues and cells. Silencing of PEG10 could inhibit proliferation, migration, and invasion via regulating the miR-449a/RPS2 axis in NB cells. These findings provided strong evidence that PEG10 might be an available target for NB management.

## Data Availability

The datasets used and analyzed during the current study are available from the corresponding author on reasonable request.

## Highlight

1. The levels of PEG10 were increased in neuroblastoma tissues and cells.

2. PEG10 promoted neuroblastoma cell proliferation, migration, and invasion.

3. PEG10 silencing inhibited neuroblastoma cell proliferation and invasion via the miR-449a/RPS2 axis.
